# Association Mapping of Physiological and Morphological Traits Related to Crop Development under Contrasting Nitrogen Inputs in a Diverse Set of Potato Cultivars

**DOI:** 10.3390/plants10081727

**Published:** 2021-08-20

**Authors:** Cesar A. Ospina Nieto, Edith T. Lammerts van Bueren, Sjefke Allefs, Peter G. Vos, Gerard van der Linden, Chris A. Maliepaard, Paul C. Struik

**Affiliations:** 1 Centre for Crop Systems Analysis, Wageningen University, P.O. Box 430, 6700 AK Wageningen, The Netherlands; aldebarac@hotmail.com; 2 Wageningen UR Plant Breeding, P.O. Box 386, 6700 AJ Wageningen, The Netherlands; edith.lammertsvanbueren@wur.nl (E.T.L.v.B.); peter.vos@gmail.com (P.G.V.); gerard.vanderlinden@wur.nl (G.v.d.L.); chris.maliepaard@wur.nl (C.A.M.); 3 Agrico Research, Burchtweg 17, 8314 PP Bant, The Netherlands; S.Allefs@agrico.nl

**Keywords:** canopy development, genome-wide association study, maturity type, nitrogen use efficiency, *Solanum tuberosum*

## Abstract

Ample nitrogen (N) is required for potato production, but its use efficiency is low. N supply strongly interacts with maturity type of the cultivar grown. We assessed whether variation among 189 cultivars grown with 75 or 185 kg available N/ha in 2 years would allow detecting quantitative trait loci (QTLs) for relevant traits. Using phenotypic data, we estimated various traits and carried out a genome-wide association study (GWAS) with kinship correction. Twenty-four traits and 10,747 markers based on single-nucleotide polymorphisms from a 20K Infinium array for 169 cultivars were combined in the analysis. N level affected most traits and their interrelations and influenced the detection of marker–trait associations; some were N-dependent, others were detected at both N levels. Ninety percent of the latter accumulated on a hotspot on Chromosome 5. Chromosomes 2 and 4 also contained regions with multiple associations. After correcting for maturity, the number of QTLs detected was much lower, especially of those common to both N levels; however, interestingly, the region on Chromosome 2 accumulated several QTLs. There is scope for marker-assisted selection for maturity, with the main purpose of improving characteristics within a narrow range of maturity types, in order to break the strong links between maturity type and traits like N use efficiency.

## 1. Introduction

In potato cropping, farmers often abundantly apply nitrogen (N) fertilizer to ensure profits because potato plants are highly responsive to extra N [1]. This practice reduces the nitrogen use efficiency (NUE) of the crop [2,3], which is already rather low because of a shallow root system and the common cultivation in sandy soils. Leaching of excess N causes eutrophication of ground and surface water and is, therefore, a serious threat to the environment in potato production areas. Governmental regulations limiting the N supply have been installed, and these make it necessary to improve NUE at lower levels of input. Moreover, the N fertilizer regulations are specified for maturity types, at least in the Netherlands, emphasizing the need to incorporate the effects of maturity type in NUE studies.

Effects of nitrogen availability on above-ground and below-ground crop development have been widely studied. High N input increases individual leaf size and leaf longevity [4,5] and promotes branching [6], thus supporting a sustained leaf production, which enlarges the period of full soil cover (SC) [7,8]. Therefore, the crop intercepts more solar radiation and accumulates more dry matter with more N [9], all resulting in higher yield, but lower NUE [2,3].

An increase in nitrate available for the plant was reported to lead not only to a reduction in the proportion of dry matter allocated to roots but also to an increase in the total root surface and root length [10]. Differences in N uptake efficiency of two cultivars were attributed to the general differences in root morphology and to a particular N response of the cultivars [10]. Moreover, high N availability tends to suppress or delay tuber bulking and affect dry matter partitioning between haulm and tubers [5]. Additionally, N input also affects tuber size and quality parameters, including tuber dry matter content, tuber starch content, tuber protein content, tuber nitrate content, and processing quality [4,11]. With more N, the proportion of large tubers was shown to increase and the fry color was shown to become darker, while the effect on tuber dry matter content was ambiguous [4,12,13].

Studies on canopy cover have shown a high correlation among the ability of genotypes to intercept photosynthetically active radiation, to change resource use efficiency, and to create tuber yield [9,14,15]. Khan [16] and Khan et al. [17,18] studied the canopy development (CDv) of potato using an eco-physiological model in which canopy growth is a function of thermal time, following the beta function as described by Yin et al. [19]. This methodology allows the dissection of the complex trait of canopy growth into model parameters with biological meaning [20,21,22]. The analysis of the curve parameters as new traits allowed capturing differences in N response among cultivars and maturity types, as well as among cultivars within the same maturity class, facilitating the understanding of the N effects on different stages of CDv [3,16,17,18,23]. Furthermore, those canopy cover traits had high heritabilities [16], and some of them showed high correlations with yield, maturity, and N content, allowing an interpretation of how the NUE of potato is affected and showing potential as selection criteria for NUE [3,11,16]. In addition, these parameters were found to be related to genetic factors (quantitative trait loci; QTLs) that act during development of the canopy cover and are probably involved in the underlying physiological processes [24]. The combination of this eco-physiological growth model and QTL analysis is a two-step approach, where the first step is to model the complex trait identifying biologically relevant parameters demonstrating genetic variation, and the second step is to use these parameters as new traits to find QTLs [25,26]. In potato, the two-step procedure was used to study the dynamics of senescence and the adaptation in potato under different day lengths [27], and to identify QTLs related to canopy cover parameters [16], as well as QTLs related to the N effects on the canopy cover parameters in a diploid mapping population [24]. 

In recent years, association mapping approaches have become increasingly popular for genetic studies, offering a series of advantages that include higher mapping resolution and results that are applicable to a wider genetic background [28]. Association mapping (AM) identifies QTLs by examining marker–trait associations resulting from linkage disequilibrium (LD) between markers and trait functional polymorphisms across a set of diverse germplasms [28]. AM copes better with tetraploid, noninbred crops, such as potato [29], than linkage analysis using segregating biparental tetraploid populations for which tetrasomic inheritance is complicated [30]. AM can detect QTLs at the tetraploid level within a genetic background that is more representative of the breeding germplasm of the crop [31]. Moreover, AM procedures can effectively compare a greater portion of the variation within a species while the traditional linkage analysis is limited to the variation in the two parents of the segregating population [32]. However, in AM, it is important to consider the effect of population structure and/or kinship because any association may partially be caused by population admixture, leading to plausible but false marker–trait associations [28,32,33]. The success of association mapping efforts depends on the possibilities of separating LD due to genetic linkage from LD resulting from other causes [31].

Several papers reported on association mapping studies in tetraploid potato. Gebhardt et al. [34] and Simko [35] reported markers associated with resistance to diseases using a form of *t*-test. Malosetti et al. [31] proposed an AM approach based on mixed models with attention for the incorporation of the relationships between genotypes, whether induced by pedigree, population substructure, or otherwise. D’hoop et al. [36] applied a simple regression-based AM approach for quality traits in potato with promising results for these traits in a large set of tetraploid cultivars. In this paper, we combine the model for canopy development and association analysis to study the genetic basis of developmental physiological and agronomic traits in relation to contrasting N levels. We performed genome-wide AM for canopy development parameters and agronomic traits in a set of 169 tetraploid potato cultivars. Our cultivar set was phenotyped and studied for canopy development under contrasting N levels; in addition to effects of environmental factors, we observed genetic variation in the model traits and in agronomic traits [3], as required for a genome-wide association analysis. Moreover, we analyzed N-dependence of the detected QTLs to show the genetic response to such an important factor and to show the usefulness of the canopy development analysis in combination with genetics studies.

In summary, our objectives were to carry out a genetic analysis on model-based phenotypic variables associated with above-ground and below-ground development. Since these variables are known to be sensitive to nitrogen supply, the analysis was carried out using data of phenotyping experiments with two contrasting nitrogen input levels. Similarly, these variables vary among maturity types and show strong interactions between maturity type and nitrogen supply; we were, therefore, interested in the genetic background of the cultivar × nitrogen interaction and whether marker–trait associations were consistent across maturity types and nitrogen supplies.

## 2. Results

### 2.1. Phenotypic Data

**Figure 1:**
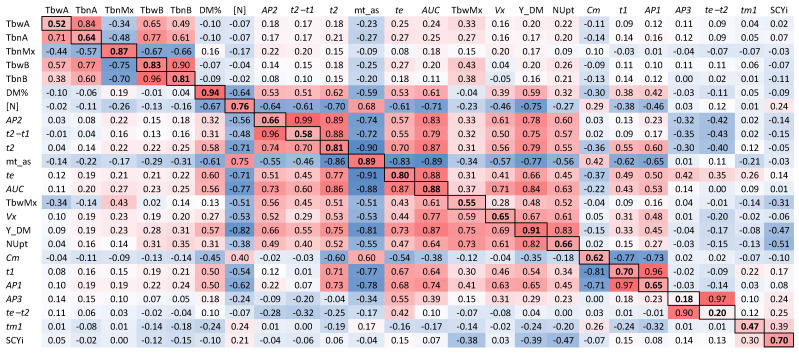
Heatmap of Pearson correlations between traits using genotypic values: Correlations at high N are in the upper triangle; correlations at low N are in the lower triangle. The diagonal contains the correlations for each trait between high and low N. The trait order was defined by cluster analysis using the high-N correlation matrix. For an explanation of the acronyms of the traits, see Section 5.5 and Section 5.6.

The phenotypic dataset used for the association analysis was collected and analyzed as described by Ospina et al. [3]. Here, we investigated the genotypic correlations among all traits at high N, low N, and across N input levels (Figure 1). As the correlations were calculated on the basis of estimated genotypic main effect values, these are effectively genetic correlations (i.e., after excluding the effects due to other terms in the model; see Section 5.7). A summary of data for all traits per N level is included in Appendix A. For an explanation of the acronyms of the traits, see Section 5.5 and Section 5.6.

**Figure 2:**
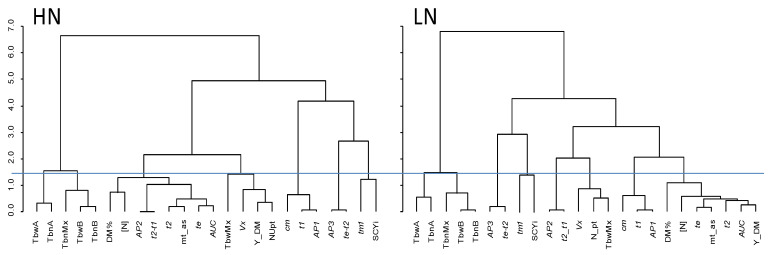
Hierarchical cluster analysis of the traits at both N levels (HN and LN, high and low N, respectively); 1 minus the absolute correlation between each pair of traits was considered as a measure of dissimilarity. For an explanation of the acronyms of the traits, see Section 5.5 and Section 5.6. The blue line is an arbitrary threshold at which clusters of traits resulting from the two dendrograms were compared.

Genetic correlation matrices for both high and low N conditions are shown in Figure 1 (corresponding to the right upper and left lower triangles, respectively). A Mantel test to compare the two genetic correlation matrices showed a high and positive association between the Pearson correlations under high and low N (Mantel test *r* = 0.9384, significance = 0.001), which was also reflected in similar grouping of the traits in a hierarchical clustering using Pearson correlation as a similarity measure (see dendrograms in Figure 2). However, there were slight differences between the clustering results at each N level.

Looking at one trait at a time, the highest correlation at each N level was between the same traits. Exceptions were *tm1*, *t2*, *te*, TbwMx, TbnA, and mt_as. For each of these traits, the highest correlation (to whichever other trait) was different for low N and high N. In general, with the diagonal in the correlation matrices excluded, there were 169 out of 552 combinations with absolute correlation coefficients lower than 0.4 at both N levels (using 0.4 as threshold, i.e., equivalent to a significance level smaller than 3.5 × 10^−10^ to prove that the correlation was different from 0, to describe the matrix; Figure 1). Additionally, there were more pairwise correlations with absolute values higher than 0.4 at low N than at high N, showing the overall effect of N on trait relationships.

The diagonal of the matrix (Figure 1) shows the correlation coefficients between N levels for each trait. *AP3* and *te–t2* were the least consistent traits across N levels with very low values (0.18 and 0.20, respectively). The traits showing the highest positive correlations between N levels were DM%, Y_DM, mt_as, *AUC*, and TbnMx. Thus, the expected interaction of these traits with N level across the cultivar set was lowest. 

Looking at hierarchical clustering of traits (Figure 2), yield (Y_DM) was grouped closer (more similar) to the CDv parameters *AUC, t2,* and *te*, as well as to maturity mt_as, [N], and DM%, at low N than at high N. At high N, NUpt, *Vx*, and TbwMx were closer to yield. Furthermore, there were five traits clustering together under both N conditions. This group included all traits from tuber size-weight and size-number distribution but not TbwMx (left-hand side of both HN and LN hierarchical trees in Figure 2), all being highly correlated to each other, as they describe the same phenomenon: tuber size distribution.

**Figure 3:**
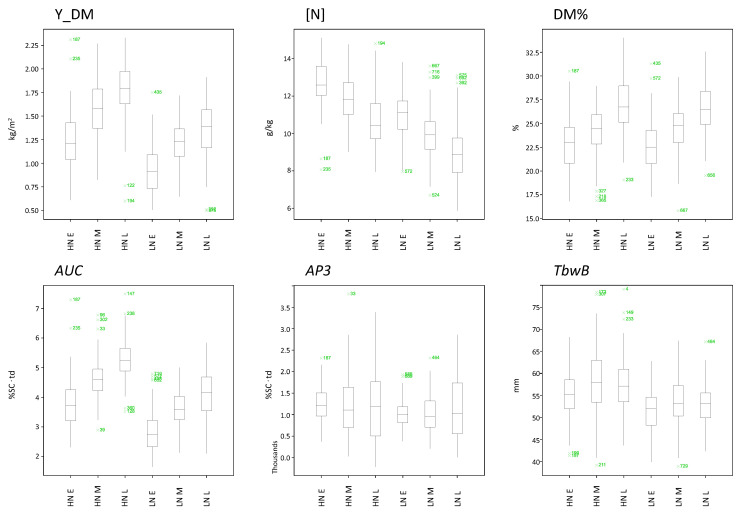
Boxplots of some traits to illustrate the data variation between maturity groups and nitrogen levels. The grouping factor on the *x*-axes is a combination of N level and maturity group as follows: HN (high nitrogen) in combination with the maturity group HN E (early), HN M (middle), and HN L (late). LN (low nitrogen) in combination with the maturity group LN E (early), LN M (middle), and LN L (late). The traits included are as follows: Y_DM, yield dry matter; [N], nitrogen content; DM%, dry matter percentage in tubers; *AUC*, area under the curve for canopy development; *AP3*, area under the curve for the Phase 3 of CDv (canopy decay); TbwB, size tuber class where the maximum tuber weight occurs. Green “×” symbols followed by number codes represent outliers.

The box plots (Figure 3) illustrate the variation among maturity groups at both N levels for selected traits. The differences among maturity groups (Mt) were not significant for the traits *tm1*, *AP3*, SCYi, and TbwA (Appendix B; *AP3* is shown as an example in Figure 3). [N] is an example of a trait for which the differences between Mt were significant, supported by a positive and high correlation with the maturity assessment (mt_as). *AUC* and yield were also significantly affected by Mt, showing a negative correlation with mt_as.

The effect of N level was significant for most traits except for *AP1*, *te–t2*, DM%, SCYi, and TbnMx (see also Appendix B). In Figure 3, DM% is shown as an example of a trait that was unaffected by N levels. [N], *AUC*, and Y_DM were strongly influenced by N input. Moreover, *AUC*, which is a parameter accumulating temporal and spatial progression of canopy development (which is directly linked to the amount of intercepted light and, therefore, photosynthetic potential over the whole growth cycle) was highly positively correlated with yield at both N levels. More N input promoted vegetative growth and prolonged the growing period, and both effects may support higher yields, provided the growing season is long enough for the potato tuber yield to benefit from the prolonged canopy development. Lastly, the tuber size with maximum weight (TbwB) was significantly affected by N but not by Mt, and this trait is a representative of a group of traits that behaved consistently different than other traits included in the analysis at both N levels (Figure 2).

### 2.2. Association Mapping

The association mapping was performed with kinship correction to minimize false positive associations. The marker–trait associations reported here had −log_10_(*p*) values higher than 4 and explained at least 10% of the variance. The results of the association mapping are presented as marker–trait associations to generally describe the output of the analysis, to have an overall impression of the N level effect on the detection of associations in our dataset and to assess the colocalization of association with markers related to maturity. QTLs were defined using a linkage disequilibrium window of 8 Mb as mentioned in Section 5.

An overall summary is shown in Table 1A. The majority of the marker–trait associations were year-dependent, with only 166 associations (out of 950) detected in both years, reflecting a strong influence of environmental conditions. We focused on marker–trait associations detected in both years to compare the results for high and low N levels. In general, more marker–trait associations were detected under high N input than under low N.

**Figure 4:**
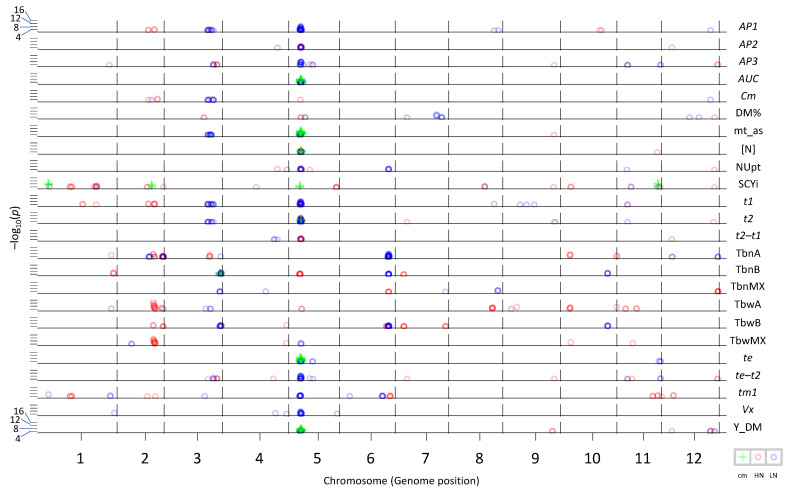
Visualization of genome-wide association analysis (data not corrected for maturity); cm = common associations between N levels (green +), HN = associations at high nitrogen (red circle), and LN = associations at low nitrogen (blue circle). All traits are included, and only associations detected in both years with a −log_10_(*p*) value > 4 are shown. A higher intensity of the color corresponds to a higher number of marker–trait associations located in close proximity of each other.

Overall, 20 traits showed associations that were present in both years (irrespective of the N levels). A QTL for maturity assessment (mt_as) in our experiment was detected in the region on Chromosome 5, reported as maturity-related in the literature [37,38,39,40,41]. This region was an association hotspot with 11 traits (*AP1, AP2, AUC*, mt_as, [N], *t1*, *t2*, *t2–t1*, *te*, *te–t2*, and Y_DM). On Chromosome 2, there was another region accumulating associations for six traits (SCYi, *AP1*, *t1*, TbnA, TbwA, and TbwMx), while there was a region on Chromosome 3 with markers associated with mt_as, TbnA, TbnB, TbnMx, and TbwB (Figure 4).

Trait associations detected at both N levels (Table 1A, common to high N and low N) were considered N-independent associations. Eighty-eight percent of these associations were with markers also associated with mt_as within a window of 8 Mb (see Section 5) on Chromosomes 5 or 3. Eleven QTLs for eight traits were N-independent (Table 2). Six of these QTLs (for *AP2*, *AUC*, [N], *t2*, *te*, and *te–t2)* were located on Chromosome 5. The other two traits were SCYi (with QTLs on Chromosomes 1, 2, and 11) and TbnB (with a QTL on Chromosome 3).

Marker–trait associations detected at only one N level were considered N-dependent associations. At high N, 45% of these associations involved mt_as-associated markers (on Chromosomes 5 and 3), all within the LD window of 8 Mb. Eleven traits with 24 QTLs were exclusively detected at high N (Table 2), and nine of these traits (*AP1*, SCYi, *t1, t2–t1* TbnMx, TbwA, TbwMx, *tm1*, and Y_DM) did not have QTLs colocalizing with maturity assessment (Table 1A and Table 2).

At low N, 27.7% of the N-dependent marker–trait associations colocalized with mt_as (Table 1A). Seven traits (DM%, *t2–t1,* TbnA, TbnB, TbnMx, TbwB, and TbwMx) showed a total of 11 QTLs with markers not colocalizing with mt_as (Table 2). Moreover there was an N-dependent QTL for mt_as detected at low N on Chromosome 3 and a QTL for *te–t2* detected at low N but colocalizing with mt_as on Chromosome 5. More QTLs were detected at high N level than at low N level, and even fewer QTLs were detected at both N levels (Table 2).

### 2.3. Association after Maturity Correction

Maturity had a strong effect on the traits measured at both high and low N, and the genomic region associated with maturity type was related to most of the traits measured. To gain more insight into the effect of maturity and to allow detection of maturity-independent QTLs, we corrected for the effect of the maturity classes (i.e., the differences in the means of the trait values between the maturity classes), effectively equalizing the trait means per maturity group. The relative differences between genotypes were maintained within a maturity group but corrected when comparing genotypes across maturity groups.

The total number of associations detected with the phenotypic data corrected for the maturity main effects (CD) was 348, with 181 markers (Table 1B), much lower than with the noncorrected data (NCD) (Table 1A). The number of associations consistently found in both years at either N level was almost half compared with the NCD, but the number of markers involved was very similar (74 compared with 66 for the NCD and CD, respectively). This is because most of the trait associations colocalizing with the maturity assessment in the NCD disappeared after correction, as expected. The number of marker–trait associations common to both N levels after the correction was only eight, involving three traits (SCYi, TbnB, and DM%).

There were 24 QTLs for 11 traits commonly detected with both datasets (CD and NCD) (Table 3), with more QTLs detected at high N than at low N (13 and six, respectively), while five QTLs were common to both N levels. A QTL for SCYi on Chromosome 5 (no QTL detected for mt_as at this position) was consistently detected at both N levels in both analyses (with CD and NCD). Furthermore, there were 17 QTLs for 11 traits detected only after the maturity correction. Three QTLs were N-independent (for DM%, *te–t2,* and [N]), while 14 QTLs were N-dependent with seven QTLs at high N (for TbnA, SCYi, DM%, TbwMX, SCYi, and mt_as) and seven QTLs at low N (for *tm1, t1, Vx, AP2, AP3*, NUpt, and TbnA). There were regions accumulating QTLs for three or more traits on Chromosomes 3, 4, and 12.

## 3. Discussion

In this study, we combined canopy development modeling with an association mapping analysis to reveal the genetic basis of developmental, physiological, and agronomic traits with varying N availability. We applied established methodologies as used by D’hoop [36,42]. The association analysis was done after correction for relatedness, which is the accepted standard because it decreases the probability of false positives [31,43]. In potato, D’hoop et al. [42] showed an increased level of LD within specific cultivar groups demonstrating the importance of correcting for relatedness.

Canopy cover of potato shows a very large genetic variation [3,17,18,44,45,46] and a large genotype × environment interaction [47,48,49]. It is controlled by multiple interacting genes, each having only a relatively small effect [27], like in many other crops [50,51,52,53]. Moreover, the genotype × environment interaction is strongly influenced by the maturity type of the cultivar [17,18]. However, the impact of the genotype × environment interaction on the various components of canopy cover over time is variable [3,17]. These effects come about through the impact of environmental factors on stem development (stem number, stem branching, and sympodial growth), leaf appearance, leaf expansion, and leaf senescence [54]. In the specific case of nitrogen supply, the interaction between the cultivar and nitrogen supply affects the number of branches (either the lower lateral branches or the sympodial branches at the top of the stem), the number of leaves on the main stem and on the different types of branches, the rate and duration of leaf expansion (resulting in the final size of the individual leaves), the duration of the life span of the leaf, and the rate of leaf senescence [55]. In close interaction with the maturity type [3], nitrogen supply supports a rapid development of the canopy early in the growing season and a long duration of canopy cover throughout the remainder of the season, thus enabling an advanced, enhanced, and prolonged light interception, allowing high tuber yields [3,17,56].

Our results showed effects of N levels on the relationship between traits based on the genetic correlation (Figure 2), similar to the results based on phenotypic correlation for both N levels reported by Ospina et al. [3]. We demonstrated the effect of N input on canopy development and yield traits (Figure 3), as well as the strong contribution of maturity type, which is the major factor determining development, to the genetic variation. The genetic variation resulted in QTLs consistently detected in both years at both N levels for 20 of the 24 traits included in this study. Many of these QTLs accumulated in a single region on Chromosome 5 that is known to be linked to maturity type as shown by Kloosterman et al. [40], who identified an allelic variation of the CDF1 (cycling DOF factor) gene at this locus which strongly influences phenology, plant maturity, and onset of tuberization, reflecting the importance of this region for quantitative developmental traits.

Effects of nitrogen availability on potato development were reported by many authors [2,3,4,5,6,9,11,12,13,14,16]. As stated above, in general, more available nitrogen advances, enhances, and prolongs canopy cover as a result of improved haulm growth [57], as well as the initiation of more leaves with a longer life span [4]. Our canopy development model describes this elegantly and in a quantitative way with biologically meaningful parameters, thus illustrating the genotype × environment interaction in an analytical way. The three phases of canopy development (see Section 5.6) responded to N with cultivars having a faster buildup phase of the canopy, resulting in a shorter time to reach maximum coverage (*t1)* and a higher maximum cover (*Vx*) for a longer period (*t2–t1*) at high N input, all resulting in higher photosynthetic potential [3,14].

Most traits included in this study had relatively high genetic correlations between high and low nitrogen conditions, except for *AP3, te–t2* (Phase 3 of CDv), and *t1*. These high correlations reflect the consistency of the genotypic behavior under varying N availability, at least for canopy development parameters associated with the period of maximum canopy cover. Phase 3 of CDv was difficult to phenotype precisely due to the senescence process itself, which starts with yellow leaves until an uncertain point when the canopy collapses. The yellowness could start early if conditions are not favorable but the crop continues to take up nitrogen. On the other hand, wind and rain can accelerate the collapse and those factors are difficult to predict. Therefore, Phase 3 parameters showed the largest random error, explaining the low heritabilities of *AP3* and *te–t2* [3]. The relationships between some of the traits based on their genetic correlation coefficients were slightly different between N input levels. For instance, yield has an absolute correlation with *AUC* higher at low N than at high N, as a result of changes in the relationship with other traits. Under high N input, there are no nutrient constraints for canopy development leading to an expansion of the duration of the potato growth phases [3,14]. However, it is known that, with high N input, important traits determining yield such as leaf area index (LAI) and radiation use efficiency (RUE) are positively affected [24]. LAI continues to increase even when the soil coverage is 100% [9,58]; the maximum coverage is also reached faster and sustained longer at high nitrogen input [3]. Therefore, although yield and *AUC* are highly correlated under both N conditions, the contribution of LAI and RUE under high N may not be fully captured by the *AUC.* This could also be reflected in the QTLs detected; QTLs common to both N levels for yield and *AUC* were found on Chromosome 5 (Table 2) and colocalized with QTLs for maturity (mt_as), while QTLs exclusively detected at high N for yield were located on Chromosomes 9 and 12. A possible explanation is that the latter two might be associated with the contribution of RUE and/or LAI to yield.

Genomic regions with possible pleiotropic effects were detected on Chromosomes 2, 5, and 6 (Figure 4). The QTL hotspot on Chromosome 5 was the most noticeable, accumulating QTLs for 50% of the traits on this maturity-related region, as similarly shown by Hurtado-Lopez et al. [39] with developmental traits related to senescence and flowering and with plant height. Most of the traits with QTLs in this region were highly correlated with maturity assessed in our trials (mt_as), emphasizing the importance of maturity and the genomic region on Chromosome 5 for crop development. Moreover, as a general remark, the colocalization of QTLs was mostly determined by the correlation between traits. Furthermore, there was an N dependency of some QTLs for several traits. The region on Chromosome 2 accumulated QTLs for six traits (*AP1*, *t1*, TbnA, TbwA, TbwMX, and SCYi) at high N input, while the region on Chromosome 6 was related to four traits (with QTLs for TbnB, TbnA, TbwB, and *tm1*) at low N input. This shows the strong effect of available N on the genetic response, as well as its complexity.

Regarding the N-dependent QTLs, at high N input, more QTLs involving more traits were detected than at low N input, along with a higher percentage of marker–trait associations on Chromosome 5. Gallais and Hirel [59] found in maize more QTLs for some traits at high N input than at low N input (vegetative development, N uptake, and yield components), while, for other traits, it was the opposite (N utilization efficiency and protein content). This is a reflection of the difference in the expression of the genetic variability between high and low N input that may be trait-dependent. The trait × nitrogen interaction was translated into a QTL × nitrogen interaction in those studies, as well as in our study.

N-independent associations were mostly located at the maturity locus on Chromosome 5 (data uncorrected for maturity). Khan [16] used a similar phenotyping approach to study potato canopy development and reported a major QTL hotspot on Chromosome 5 in a diploid biparental population (SH × RH) affecting all parameters of the canopy cover curve in several environments. Ospina [24], using the same diploid biparental mapping population, reported the same QTL region on Chromosome 5 at both high and low nitrogen levels. In addition, QTLs for growth and yield traits in this region were found in drought tolerance QTL mapping in the greenhouse of the C × E diploid mapping population [60] and in multiple environments for the same population [27,39]. Therefore, the overall and predominant effect of maturity on canopy development and on yield appears to be stable across different environments, nitrogen conditions, and populations.

N-independent QTLs different from those of the maturity locus on Chromosome 5 were found only for SCYi and TbnB (Figure 4). These two traits were not correlated with the maturity assessment (mt_as) (Figure 1). For SCYi, there were N-independent QTLs on Chromosomes 1, 2, 5, and 11, while, in the diploid mapping population, SH × RH [24], N-independent QTLs were found on Chromosomes 5 and 10 (referred to as linkage groups V and X in the multitrait QTL analysis [24]). This might suggest that the genetic background, as well as the population type, influences the genomic regions related to a trait. For TbnB, we found N-independent QTLs, as well as a QTL at low N level, on Chromosome 3. Schönhals [61] also found associations for tuber number on this chromosome (as well as on Chromosomes 1, 5, and 6), using markers for candidate genes that were functionally related to tuber yield and starch. A comparison of the results with previous reports is difficult because different markers were used in different populations. For the markers used in the detection of QTLs with the SH × RH diploid biparental population by Ospina [24], there were no physical positions available (these makers were not used in this association analysis), while, for the SNPs used here in the association mapping, there are no genetic positions known on the SH × RH genetic map.

After the maturity correction, the number of N-independent marker–trait associations was drastically reduced. Since most of the traits were maturity-related, the maturity correction was expected to have a strong impact on the detection of QTLs. Only the N-independent QTLs for SCYi and TbnB remained after the correction (these were not linked to maturity, and the traits did not correlate with maturity). Similarly, D’hoop et al. [62] showed the impact of maturity. In their phenotypic analysis, the presence or absence of maturity as a term in the model influenced the genotypic effects for two traits studied, underwater weight and maturity trait (both traits are physiologically correlated), but not for the majority of quality traits, which were not correlated with maturity. Their association analysis using maturity-corrected values in a model with a correction for relatedness showed a reduction in the number of marker–trait associations detected for these two traits (underwater weight and maturity), while, for other quality traits, there was no clear trend [63].

The maturity correction of our data using predefined information of the cultivars (the maturity grouping factor) was effective in removing the maturity effect, although it might have affected the detection of association, most probably reducing the power since the overall variation was reduced. However, it allowed detection of new QTLs (Table 3) that did not colocalize in the main region related to maturity on Chromosome 5. For instance, a new N-independent QTL for DM% on Chromosome 3 was detected. This QTL was expected to be N-independent since the DM% trait did not have a significant nitrogen effect (Appendix B). The results with the diploid population SH × RH [24] also showed a QTL for DM% on Chromosome 3. In general, the maturity dependency of some QTLs resulted from the physiological relationship of canopy development traits and agronomic traits with maturity (these relationships were discussed by [3,24]). Kloosterman et al. [40] identified the causal gene within the Chromosome 5 maturity locus. Allelic variation of the CDF1 (cycling DOF factor) gene at this locus strongly influences phenology, plant maturity, and onset of tuberization. The CDF1 gene has a great effect on the plant life-cycle length by acting as a mediator between photoperiod and the tuberization signal. This major effect acts on several processes of the plant, resulting in a strong linkage between maturity and traits related to CDv and yield. Khan [16] also mentioned the dependency of tuber yield on its components (especially tuber bulking parameters and CDv traits), as these are physiologically and surely genetically related; genotypes with higher tuber bulking rates show limited haulm growth and canopy duration, leading to an early maturity type [16].

We showed how genetic factors determining canopy development and yield traits in potato cultivars interact with N levels. The different QTL regions detected for a trait under contrasting N conditions may imply that the phenotypes are the result of a tradeoff between these QTL regions. The detection of N-dependent QTLs emphasizes the importance of direct selection under limiting N conditions only if the QTLs contribute to the traits of interest. The contribution of genetic factors to growth and yield is affected by N input, with different interactions between the traits under low N than under high N and, therefore, different contributions of the traits to the observed phenotype. Ospina et al. [3] mentioned that, to breed for NUE under low input, the strategy should be to select for high yield under low N and combine this with a high responsiveness to more N input. This allows for selection of better adapted genotypes in N-limiting conditions. In addition, to bypass the strong linkage with maturity that is observed for developmental traits, NUE, and yield, the selections should be done within each maturity group. Thus, the phenotyping should be made more discriminative to exploit the variation in a narrow maturity category. Additionally, the strong correlation of most of the traits mentioned with maturity can mask useful genetic variation for these traits, as exemplified in this paper. An early selection is required to increase the number of individuals in the target maturity class. This might be achieved by developing marker selection for maturity. Small differences in the existing trait will then be detectable, and selectors and breeders may be able to identify new traits and be more discriminant in their assessments for these other traits.

In general, the reports in other crops on QTL detection under contrasting N conditions have shown the great influence of environmental conditions. For example, in barley, the detection of QTLs was reported to show an extensive G × E/QTL × E interaction, with QTLs changing between years irrespective of N levels [64]. In maize, contrasting results have been reported when looking for QTLs under high and low N input. QTLs for grain composition and NUE-related traits detected at low N corresponded to QTLs detected at high N input [65], while other results showed that QTLs for NUE were only detected at low N input [66]. Hirel et al. [67] suggested that, depending on the recombinant inbred line population, the response of yield to various levels of N fertilization could be different and, thus, controlled by a different set of genes. We only reported QTLs consistently detected in both years for each N level. Therefore, our research does not directly address the G × E interaction, for which more experiments would be needed.

However, our research also demonstrated extensive G × E/QTL × E interactions, as illustrated in Table 1; the number of marker–trait associations was significantly higher across all datasets (950 marker–trait associations detected irrespective of the year) than the number of associations detected in both years (only 166 marker–trait associations detected in both years). In the context of our experimental setup, nitrogen level was the major control factor driving the differences within this very constant physical environment. The total number of marker–trait associations (*n* = 166) detected in both years could be broken down in groups that were detected at both N levels (*n* = 50), only at high N (*n* = 69), or only at low N (*n* = 47) (Table 1). Moreover, there was a strong effect of maturity type on the detection of these marker–trait associations. When data were corrected for maturity type, only 86 marker–trait associations were found, which could be broken down into three groups; eight were detected at both N levels, 48 were only detected at high N, and 30 were only detected at low N (Table 1). Therefore, the nitrogen dependency of some QTLs could be interpreted as a QTL × N interaction.

Our approach focused on contrasting N input levels using a single N application, and this is a first step to understanding the genetic factors involved in the response of potato to N. It is important to mention that fertilization practices such as split application might have an additional effect on the plant response to nitrogen, especially in relation to the different maturity types. Additionally, soil mineral N supply during the growing season is difficult to control and understand [7] since it is a dynamic factor. Goffart et al. [68] mentioned that soil mineral N supply is influenced by several predictable and unpredictable factors, such as weather conditions, chemical and physical soil properties, type and evolution of organic matter previously incorporated in the soil, cultural practices, maturity type of the cultivar, and crop duration. This N dynamic in the soil could result in different levels of available N. This difference will affect the crop development response of the cultivar and, thus, the variation of the traits, thereby affecting the consistency in the detection of QTLs or marker–trait associations.

Lastly, the understanding of the influence of an intrinsic major genotypic factor such as maturity type is valuable to refine breeding strategies, as well as to develop cultivars suitable to low N input or otherwise limiting conditions. Furthermore, the results presented here suggest that breeding schemes should be done within maturity groups, with the main idea of improving characteristics that are highly influenced by maturity, such as DM%, N content, and NUE, within a maturity group.

## 4. Conclusions

We used a detailed phenotyping dataset based on a 2-year experiment with 169 cultivars or other genetic material, grown at two levels of nitrogen in an otherwise very uniform physical environment, and we analyzed that dataset using a dynamic canopy cover model with biologically meaningful parameters. Cultivar-specific parameter values of that model and the tuber growth characteristics model were associated with markers from a 20K Infinium array. Twenty-four traits and 10,747 SNP markers were combined in a GWAS with kinship correction. Nitrogen had a strong effect on most traits and on the correlations between these traits. Nitrogen also strongly affected the detection of marker–trait associations. We could show that some associations were detected at both nitrogen levels, whereas others were only found at a low or high nitrogen level. However, correction for maturity type proved to be essential for the interpretation of the data. After correcting for maturity, the number of QTLs detected became much lower, especially for those that were common to both N levels; however, interestingly, a region on Chromosome 2 accumulated several QTLs. Apparently, there are strong links between maturity type and traits associated with nitrogen husbandry of the potato crop.

## 5. Materials and Methods

The experimental design, data collection, and processing to generate the phenotypic information used in this paper were described in detail by Ospina et al. [3]. Therefore, a brief description suffices here.

### 5.1. Location and Planting Material

Experiments were carried out at the Agrico research and breeding station (Bant, Flevoland, The Netherlands), in 2009 and 2010. We used a set of 189 cultivars representing a wide diversity of potato cultivars commonly grown in Europe, ancestors, and progenitor clones (Appendix C). The set has been extensively used for association studies of quality traits, as described by D’hoop et al. [36,42].

### 5.2. Experimental Design and Treatments

In both experiments, two N levels were implemented: (i) high N, with 180 kg available N/ha (soil N and fertilizer N combined) as a standard conventional N input level, and (ii) low N, with 75 kg available N/ha as the low input variant. The amount of fertilizer required was calculated on the basis of a soil analysis done at the beginning of the growing season. Fertilizer application was split into two. A basic fertilizer treatment was applied just after planting (NPK) on the whole experimental field to reach the amount for low N. A second amount was applied to the high-N plots only, before the final ridging, using dolomite ammonium nitrate (DAN, 27–0–0). P and K were abundantly available for potato crop growth in both N treatments.

The experimental design was an unbalanced split-plot design, with 16 plants per genotype per field plot, with treatments (N) as whole plots (with no replicates), maturity groups as subplots randomized within whole plots, and cultivars nested and randomized within maturity subplots. An additional 16 (2009) or 20 (2010) field plots with a reference cultivar were planted at random across the field to estimate the plot-to-plot environmental variation without confounding cultivar variation.

### 5.3. Data Collection

Emergence date was estimated per plot as the first date when more than 50% of the plants in the plot had emerged (i.e., first leaf visible). The percentage of soil cover (SC) was assessed weekly over three plants per plot (same three plants) all through the growing season from emergence until harvest. Maturity was scored using a scale to assess the progress of senescence (modified from [37]) in which 1 = green canopy with the first flower buds, 2 = green haulm with abundant flowers, 3 = first signs of yellowness in the upper leaves, 4 = up to 25% of the plant with yellow leaves, 5 = up to 50% of the plant with yellow leaves or lost leaves, 6 = up to 75% as in 5, 7 = up to 90% of the plant yellowed or without leaves, and 8 = entire haulm brown or dead. This assessment is referred to as maturity assessment (mt_as) to avoid confusion with the maturity index used to form maturity groups as blocking factor (Mt).

### 5.4. Final Harvest

The final harvest took place as late as possible to allow late cultivars to complete their cycle. The whole experiment was harvested at once. Sixteen plants were harvested per plot, and the following tuber traits were assessed: (A) total tuber fresh weight; (B) tuber size and weight distribution, for which six size classes were included (0–30 mm, 30–40 mm, 40–50 mm, 50–60 mm, 60–70 mm, and >70 mm), recording the tuber number and tuber weight for each class; (C) tuber number per meter (obtained for the class 50–60 mm); (D) dry matter percentage (DM%), as the dry weight of a sample divided by its fresh weight, expressed as a percentage, where tubers from all size classes were cut using a French fries cutting machine before drying at 70 °C for 48 h; (E) N content ([N]) in the tubers, assessed using the Kjeldahl protocol.

### 5.5. Data Processing

A canopy development model was fitted using the NOLIN procedure of SAS/STAT^®^, with percentage soil cover as the dependent variable of Beta thermal time counted from emergence day until each assessment date. Five parameters were estimated for each individual plot [16,17,18]. Four *t*-parameters were expressed in thermal days (td): *tm1* (inflection point in the growing phase of the curve), *t1* (when SC stabilized), *t2* (start of senescence), and *te* (when canopy had completely senesced). The fifth parameter, *Vx*, was the maximum SC reached with percentage soil coverage (%SC) as unit.

**Figure 5:**
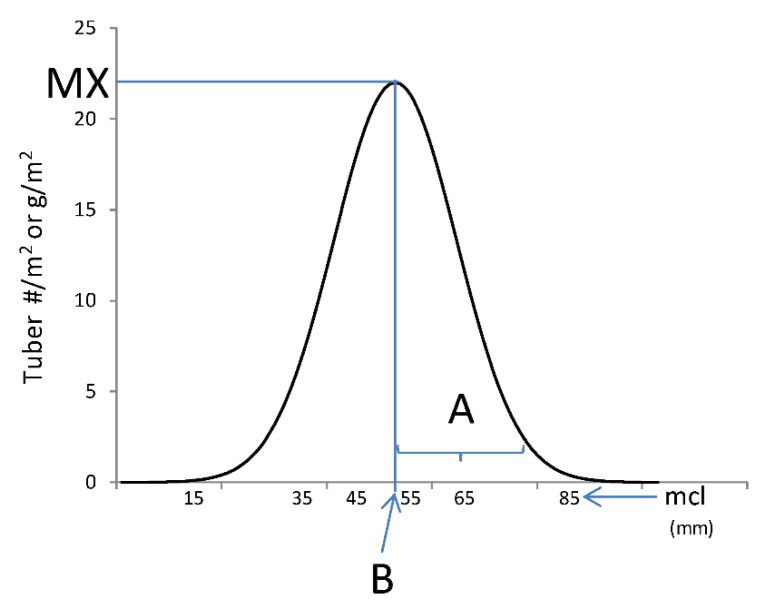
Bell-shaped curve and parameter representation of Equation (1) (Equation (1)). Parameter names are explained in the text (see Section 5.5). Tb is either Tbw or Tbn, “A” is a dispersion parameter expressing how the weights/numbers were distributed across tuber size classes, “mcl” is the average size of each tuber size class, and “B” is the average size at which the maximum (“MX”) weight/number occurs. The curve-fit parameters were named for each variable as follows: For Tbw data, TbwA, TbwB, and TbwMx; for Tbn data, TbnA, TbnB, and TbnMx.

A bell-shaped curve was fitted per plot for tuber weight and tuber number datasets separately (Tbw and Tbn respectively) to describe their distribution. Three parameters were estimated for each dataset following Equation (1) (Figure 5).

(1) $$\mathrm{Tb}=\mathrm{MX}\times \exp\left(-\frac{{\left(\mathrm{mcl}-B\right)}^{2}}{A}\right)$$

### 5.6. Calculated Variables

On the basis of the parameters estimated with the CDv model, the following variables were calculated [16,17,18]: *t2*–*t1* (duration of maximum SC in td), *te*–*t2* (duration of senescence in td), *Cm* (maximum progression rate of %SC in %/td), *AP1* (area under the curve for canopy buildup phase in %.td), *AP2* (area under the curve for phase of maximum SC in %.td), *AP3* (area under the curve for senescence phase in %.td), and *AUC* (area under the curve for the entire crop cycle in %.td). In order to express the agronomic variables in a standard way, subsequent calculations and conversions were done as follows: N content ([N]) in g/kg (determined only in tubers); DM% in percentage; dry matter yield (Y_DM) in kg/m^2^, i.e., Y × DM%/100; N uptake in tuber (NUpt) in g/m^2^, i.e., Y_DM × [N]; N use efficiency (NUE) as Y_DM/(N input) in kg/g; N utilization efficiency (NUtE), i.e., Y_DM/NUpt, in kg/g; N uptake efficiency (NUptE; NUpt/N input in g/g); and soil coverage yield index (SCYi = *AUC*/Y_DM in %.td/(kg/m^2^)). The variables were analyzed without transformation since there were no severe violations to the assumptions required for mixed model analysis. All trait acronyms are summarized in Appendix D.

### 5.7. Statistical Analysis

Data were analyzed with the Genstat package (16th edition). The model in Equation (2) combining information of both years was used for each N level.

(2) $$Y=yr*Mt+Mt.G+\underline{yr.row}+\underline{yr.col}+\underline{E}$$

where terms joined by “*∗*” represent individual effects plus the interactions (*yr* *∗ Mt = yr + Mt + yr.Mt*), whereas terms joined by “.” represent interaction only. The term *yr* represents year, clarifying that year effects include variation due to the experimental field. The term *Mt* is the maturity group excluding control plot information. Corrections for rows and columns are the random terms (*yr.row* and *yr.col*). The term *Mt.G* represents the cultivars nested within maturity groups, since maturity is an intrinsic characteristic of each cultivar. Lastly, *E* represents the error. All random terms are underlined.

The genetic correlations between traits were estimated as the Pearson correlations based on the estimated genotypic means, BLUEs, i.e., best linear unbiased estimates (excluding all other terms in Equation (2)). In addition, in order to understand relationships between traits and to define groups of traits, a divisive hierarchical cluster analysis was carried out, i.e., the analysis was top-down, performing recursive splits when going down the hierarchy. For this hierarchical cluster analysis, we used the absolute genetic correlations between traits as a similarity measure and applied the Ward minimum-variance method. The hierarchical cluster analysis was carried out separately for the two nitrogen levels. We excluded traits for which calculation included N input level, i.e., NUE, NutE, and NUptE. Additionally, biplots were generated to visualize relationships between traits per N level, included in Appendix E.

### 5.8. Association Mapping

The analysis included the 169 potato cultivars out of the total set of 189 cultivars for which genotypic data were available. SNP data were generated using a 20K Infinium SNP array [69]. A total of 14,587 markers were successfully scored in (a maximum of) five dosage classes per SNP using fitTetra [70]. The dosage classes were nulliplex, simplex, duplex, triplex, and tetraplex depending on the number of copies of the allele being quantified (0 to 4). Only SNPs having allele frequencies greater than 5% in at least two of the dosage classes were considered. Thus, frequencies of minor alleles were ignored, and a total of 10,747 SNPs were used to perform the GWAS.

The GWAS was performed using a mixed model including a kinship matrix to account for population structure. The kinship matrix was estimated using 764 SNP markers randomly distributed over the genome, expressed as (with random terms being underlined):

*Trait (y)* = *Marker (m)* + *genotype* + *residual where var(genotype)* = *Kσ_g_^2^ and K = Kinship matrix*. (3)

Linkage disequilibrium between markers was extensively studied by Vos et al. [69] and D’hoop et al. [42] using the same cultivar set. From those studies, the linkage disequilibrium decay was estimated to be between 2 and 4 Mb. We considered LD as 4 Mb and a window of 8 Mb, i.e., the apposition of a marker ±4 Mb). For a full, detailed description of the 20K SNP array, see https://edepot.wur.nl/392278 (accessed on 20 July 2021).

The association analysis was done using fitted values for the observations, (BLUPs, i.e., best linear unbiased predictions) using the model of Equation (2). Four phenotypic datasets corresponding to combinations of both years and N levels were the input in this analysis. In Section 2, we only considered associations with a −log_10_(*p*) > 4. Then, the focus was to find marker–trait associations consistent across both years, for each N level. Next, we compared results from the two N levels defining associations detected at both N levels as common (cm), as well as those exclusive to either high N (HN) or low N (LN). The last two categories were considered N-dependent marker–trait associations.

Since maturity is known to have a strong effect on the traits considered in this study [3,16,17,18], BLUEs excluding the main effect of maturity group were calculated. From each estimated value, the effect of the maturity class was subtracted, and these maturity type-corrected BLUEs were used as input in the association analysis with population structure correction.

## Figures and Tables

**Table 1:** Number of marker–trait associations detected by genome-wide association analysis using data not corrected (**A**) and corrected (**B**) for maturity. The correction was done as explained in Section 5. The associations included fulfilled the criteria of having a −log_10_(*p*) value > 4 and explained variance >10%.

**(A) Data Not Corrected for Maturity**									
**Associations**	**Detected in at least one dataset**				**Detected in both years**			**Marker maturity related (%) ^5^**	
	**Mk_T_set ^1^**	**Mk_T ^2^**	**Mk ^3^**	**T ^4^**	**Mk_T**	**Mk**	**T**	**Mk_T**	**Mk**
**Total**	950	601	282	24	166	74	20	53.0	27.0
**Common to**									
**High N and Low N**					50	19	8	88.0	68.4
**N dependent**									
**High N**					69	42	14	44.9	31.0
**Low N**					47	33	12	27.7	33.3
**(B) Data Corrected for Maturity**									
**Associations**	**Detected in at least one dataset**				**Detected in both years**			**Marker maturity related (%)**	
	**Mk_T_set**	**Mk_T**	**Mk**	**T**	**Mk_T**	**Mk**	**T**	**Mk_T**	**Mk**
**Total**	348	233	181	24	86	67	17	2.3	3.0
**Common to**									
**High N and Low N**					8	8	3	0.0	0.0
**N dependent**									
**High N**					48	42	12	4.2	4.8
**Low N**					30	21	13	0.0	0.0

^1^ Marker–trait set association; this count considers all marker–trait associations from different datasets (there were four datasets from the combination of year and N level). ^2^ Marker–trait; here the same marker–trait association over different sets was counted as 1. ^3^ Marker is the number of markers involved in a given count of marker–trait associations. ^4^ Trait is the number of traits involved in a given count of marker–trait associations. ^5^ Percentage of markers showing association with the maturity trait (mt_as).

**Table 2:** Peak markers of QTLs consistently detected, using data uncorrected for maturity.

N Level	Trait	Chromosome	Genome Position	Marker	−log_10_(*p*)	Explained Variance (%)
**cm**	***AUC***	5	316045624	PotVar0079081	8.16	24.43
	**mt_as**	5	316045624	PotVar0079081	8.46	25.10
	***t2***	5	316307819	PotVar0080570	6.73	16.16
	**TbnB**	3	216014512	solcap_snp_c2_616	5.12	15.72
	***te***	5	316045624	PotVar0079081	7.76	23.18
	**Y_DM**	5	316611906	solcap_snp_c2_50302	6.17	17.20
	**SCYi**	1	4041250	PotVar0045583	8.27	21.18
		2	131733600	PotVar0120916	5.96	18.04
		5	314920671	PotVar0025024	5.23	15.31
		11	757973524	PotVar0112496	7.17	20.40
	**[N]**	5	316307819	PotVar0080570	6.36	19.29
**cm Total**	**8**	**11**				
**HN**	***AP1***	2	127511213	solcap_snp_c2_15749	4.61	11.28
		2	135122688	PotVar0046300	5.33	12.33
		5	315893706	PotVar0026425	5.70	14.33
	***AP2***	5	316045624	PotVar0079081	5.33	18.00
	***t1***	1	46273159	PotVar0132293	4.82	11.35
		2	127511213	solcap_snp_c2_15749	5.28	13.23
		2	135122688	PotVar0046300	5.23	12.00
		5	315893706	PotVar0026425	6.95	16.68
	***t2–t1***	5	316045624	PotVar0079081	5.16	17.23
	**TbnA**	2	134943142	PotVar0045853	4.24	10.77
		2	146198944	PotVar0002966	5.07	12.61
		3	203612959	solcap_snp_c1_3637	5.88	13.38
	**TbnMX**	12	832589670	PotVar0052600	5.08	15.99
	**TbwA**	2	134242234	PotVar0128476	9.59	19.71
	**TbwMX**	2	134943142	PotVar0045853	6.14	15.09
	***tm1***	1	32843979	PotVar0000007	4.62	13.17
		6	427042067	PotVar0040538	5.12	13.61
		11	751753201	solcap_snp_c2_44269	4.70	13.56
		11	757973524	PotVar0112496	5.35	15.86
	**Y_DM**	9	627531669	PotVar0094025	4.56	14.51
		12	823287226	PotVar0037640	4.38	12.39
	**SCYi**	1	30559567	PotVar0037260	4.65	13.53
		1	61310626	solcap_snp_c2_20888	5.04	15.92
		8	543834623	PotVar0060623	5.30	15.34
**HN Total**	**11**	**24**				
**LN**	**DM**	7	484592357	PotVar0092426	7.48	11.85
		7	490792384	solcap_snp_c2_38787	4.09	20.97
	**mt_as**	3	204691153	solcap_snp_c2_29678	4.37	14.45
	***t2–t1***	4	283407138	PotVar0116182	4.21	10.32
	**TbnA**	6	424940350	solcap_snp_c2_56145	5.44	13.99
	**TbnB**	6	425163888	PotVar0074198	4.20	13.30
		10	695881376	solcap_snp_c1_13524	5.57	11.05
	**TbnMX**	3	216014512	solcap_snp_c2_616	4.34	11.33
	**TbwB**	3	217632046	PotVar0021118	5.84	18.26
		6	424915228	PotVar0074004	5.17	15.42
		10	695881376	solcap_snp_c1_13524	5.27	11.14
	**TbwMX**	2	106818648	solcap_snp_c2_4515	4.29	11.05
	***te–t2***	5	316045624	PotVar0079081	6.53	19.22
**LN Total**	**9**	**13**				

“cm” represents N-independent QTLs, i.e., QTLs detected for both N levels. “HN” and “LN” represent N-dependent QTLs, i.e., QTLs exclusively detected at high N level or QTLs exclusively detected at low N level, respectively. For the trait acronyms, see Section 5.5 and Section 5.6. Only QTLs detected in both years are included.

**Table 3:** Peak markers of QTLs consistently detected in both years (corrected and not corrected for maturity). For the trait acronyms, see Section 5.5 and Section 5.6. N level: Whether the associations were detected at high nitrogen (HN), detected at low nitrogen (LN), or common to both nitrogen levels (cm).

					Not Corrected		Corrected	
Chromosome	Genome Position	Marker	Trait	N Level	−log_10_(*p*)	Explained Variance	−log_10_(*p*)	Explained Variance
1	4041250	PotVar0045583	SCYi	cm	8.27	21.18	9.65	22.06
1	4041250	PotVar0045583	*tm1*	LN			6.36	14.49
1	32843979	PotVar0000007	SCYi	HN	4.74	12.93	4.91	13.58
1	32843979	PotVar0000007	*tm1*	HN	4.62	13.17	4.92	13.82
1	63469625	solcap_snp_c1_9676	SCYi	HN	5.07	14.44	5.59	15.39
1	81815164	PotVar0060997	TbnA	HN			5.60	14.32
2	127511213	PotVar0060997	*t1*	HN	5.28	13.23	5.33	11.55
2	131733600	PotVar0120916	SCYi	cm	5.96	18.04	6.93	17.51
2	134242234	PotVar0128476	TbwA	HN	9.59	19.71	9.31	20.68
2	134943142	PotVar0045853	TbnA	HN	4.24	10.77	4.29	10.80
2	134943142	PotVar0045853	TbwMX	HN	6.14	15.09	6.36	15.32
2	146198944	PotVar0002966	TbnA	HN	5.07	12.61	5.67	14.50
2	146303689	PotVar0003077	SCYi	HN			4.19	10.62
3	166741184	solcap_snp_c1_15204	DM	cm			5.43	13.98
3	203612959	solcap_snp_c1_3637	TbnA	HN	5.88	13.38	5.60	12.92
3	212481799	PotVar0030333	N	HN			4.33	12.00
3	212547269	PotVar0030515	*te–t2*	LN			4.49	10.32
3	212548683	PotVar0030515	*te–t2*	HN			5.08	11.57
3	213525966	PotVar0121169	N	LN			4.61	10.65
3	216081835	solcap_snp_c1_151	TbnB	cm	5.12	15.72	5.38	16.01
3	216081835	solcap_snp_c1_151	TbnMX	LN	4.34	11.33	4.73	11.96
3	217630938	solcap_snp_c1_151	TbwB	LN	5.85	18.74	5.28	17.75
4	283533011	solcap_snp_c1_15513	*AP2*	LN			4.46	10.31
4	285470025	PotVar0088487	*Vx*	LN			4.90	11.19
4	289210701	solcap_snp_c2_39807	DM	HN			4.84	11.99
4	300280572	PotVar0015935	TbwMX	HN			5.21	10.61
5	314920671	PotVar0025024	SCYi	cm	5.23	15.31	5.83	15.50
5	360216448	PotVar0082077	SCYi	HN			4.09	12.72
6	424406145	PotVar0082077	TbwB	LN	5.02	14.67	4.46	12.57
6	424940350	solcap_snp_c2_56145	TbnA	LN	5.44	13.99	4.40	12.65
6	427042067	PotVar0040538	*tm1*	HN	5.12	13.61	5.22	13.84
8	543834623	PotVar0060623	SCYi	HN	5.30	15.34	5.52	15.66
9	624408926	PotVar0051600	*AP3*	LN			4.54	10.23
10	695881376	PotVar0051600	TbwB	LN	5.27	11.14	5.57	11.22
10	695908422	solcap_snp_c2_57635	TbnB	LN	5.57	11.15	5.97	11.16
11	719755658	solcap_snp_c2_33657	NUpt	LN			5.09	12.32
11	724842000	PotVar0058777	SCYi	HN			4.46	12.67
11	751753201	solcap_snp_c2_44269	*tm1*	HN	4.70	13.56	5.24	14.68
11	757973524	PotVar0112496	SCYi	cm	7.17	20.40	7.34	21.01
12	823269906	PotVar0037718	*t1*	LN			4.21	10.98
12	827080788	solcap_snp_c1_11644	mt_as	HN			4.58	11.04
12	832202879	PotVar0052761	TbnA	LN			4.13	10.59
12	832589670	PotVar0052600	TbnMX	HN	5.08	15.99	5.13	15.96

## Data Availability

All data presented in this study may be made available upon reasonable request.

## Appendix A. Means of Traits per Maturity Group (E: Early; M: Intermediate; L: Late), and N Levels (High Nitrogen: 180 kg Available N/ha; Low Nitrogen: 75 kg Available N/ha). The Data from Both Years (2009 or 2010) Are Combined per Nitrogen Level. For Acronyms and Units of Traits, See the Main Text or Appendix D

		**High Nitrogen**			**Low Nitrogen**	
**Trait**	**Early**	**Middle**	**Late**	**Early**	**Middle**	**Late**
[N]	12.70	11.89	10.72	11.01	9.96	9.01
*AP1*	818.5	1082.1	1238.5	789.0	1199.9	1448.9
*AP2*	1707.0	2343.5	2900.5	1016.4	1412.0	1542.0
*AP3*	1230.0	1182.6	1153.5	1010.1	1002.4	1133.2
*AUC*	3756.0	4608.5	5275.0	2815.0	3614.0	4124.5
*Cm*	7.40	7.04	6.27	5.61	4.92	4.30
DM%	22.87	24.13	27.01	22.70	24.48	26.78
mt_as	6.23	4.74	3.83	6.90	5.71	4.82
NUE	0.07	0.09	0.10	0.12	0.16	0.18
NUpt	15.60	18.57	18.80	10.12	12.02	11.82
NUptE	0.87	1.03	1.04	1.35	1.60	1.58
NUtE	0.08	0.09	0.10	0.09	0.10	0.11
SCYi	3079.5	2986.5	3053.5	3094.5	3010.0	3156.5
*t1*	19.10	22.67	24.96	20.31	26.27	30.43
*t2*	38.96	48.19	56.13	34.58	44.15	49.62
*t2–t1*	19.87	25.53	31.17	14.27	17.89	19.20
TbnA	213.05	272.40	251.25	178.05	198.75	193.75
TbnB	50.43	53.44	51.98	46.89	49.18	48.49
TbnMX	21.84	22.49	25.02	21.16	22.39	25.31
TbwA	162.65	186.90	187.00	144.25	144.60	151.40
TbwB	55.65	59.19	57.51	51.51	53.81	53.10
TbwMX	2.54	3.11	3.00	2.00	2.42	2.39
*te*	60.93	67.52	74.69	56.36	62.86	72.18
*te–t2*	21.97	19.33	18.82	21.79	18.72	22.56
*tm1*	9.26	9.58	9.73	8.24	8.02	7.63
*Vx*	85.84	92.04	92.78	71.47	79.83	78.98
Y_DM	1.25	1.58	1.78	0.94	1.22	1.34

## Appendix B. The *p*-Values for Main Factors Included in the Analysis Using Mixed Model REML; *yr*: Year, *N_lv*: Nitrogen Level, *Mt*: Maturity Groups. Interaction Terms Are Represented as Main Terms Joined by a “.”. For Acronyms of Traits, See the Main Text or Appendix D

**Trait**	***yr***	***N_lv***	***Mt***	***N_lv.Mt***	***yr.Mt***	***yr.N_lv***	***yr.N_lv.Mt***
*tm1*	<0.001	<0.001	0.822	0.006	<0.001	0.309	0.212
*t1*	<0.001	0.001	<0.001	0.002	<0.001	0.881	0.057
*Vx*	0.001	<0.001	<0.001	0.124	<0.001	0.215	<0.001
*t2*	<0.001	<0.001	<0.001	0.099	<0.001	0.064	<0.001
*te*	<0.001	<0.001	<0.001	0.320	<0.001	<0.001	0.448
*Cm*	<0.001	<0.001	<0.001	0.269	<0.001	<0.001	0.338
*AP1*	<0.001	0.224	<0.001	0.006	<0.001	0.784	0.022
*AP2*	<0.001	<0.001	<0.001	<0.001	<0.001	0.211	0.117
*AP3*	<0.001	<0.001	0.553	0.150	<0.001	<0.001	0.140
*AUC*	0.009	<0.001	<0.001	0.076	0.061	0.034	0.027
*t2–t1*	<0.001	<0.001	<0.001	<0.001	0.001	0.122	0.229
*te–t2*	<0.001	0.793	0.008	0.065	<0.001	0.013	0.003
DM%	<0.001	0.915	<0.001	0.023	0.097	0.366	0.153
Y_DM	0.023	<0.001	<0.001	<0.001	<0.001	0.334	0.149
[N]	0.004	<0.001	<0.001	0.467	<0.001	0.207	0.210
NUpt	0.735	<0.001	<0.001	0.002	<0.001	0.233	0.735
NUptE	0.777	<0.001	<0.001	0.074	<0.001	0.313	0.032
NUtE	0.009	<0.001	<0.001	0.001	<0.001	0.124	0.785
NUE	0.082	<0.001	<0.001	<0.001	<0.001	0.999	0.056
SCYi	<0.001	0.242	0.424	0.627	<0.001	0.02	0.162
TbwMX	0.045	<0.001	<0.001	0.432	<0.001	0.351	0.689
TbwB	<0.001	<0.001	0.028	0.329	<0.001	0.012	0.118
TbwA	0.987	<0.001	0.192	0.119	<0.001	0.102	0.015
TbnMX	<0.001	0.437	0.021	0.638	<0.001	0.003	0.550
TbnB	<0.001	<0.001	0.022	0.436	0.026	0.004	0.600
TbnA	<0.001	<0.001	0.015	0.080	<0.001	0.015	0.066
mt_as	0.585	<0.001	<0.001	0.021	0.606	0.993	0.648

## Appendix C. Cultivars Used in This Experiment, Based on D’hoop et al.

**P8 Code**	**Cultivar or Breeder’s Clone**	**Year of First Registration**	**Country of Origin**	**Parentage**	**Market Niche**
P80002	Abundance (Sutton’s)	1886	UK	Magnum Bonum × Fox’s seedling	Ancient cultivar
P80003	Ackersegen	1929	GER	HINDENBURG × ALLERFRÜHESTE GELBE	Ancient cultivar
P80004	Adirondack	1881	USA	Peachblow × Peachblow	Ancient cultivar
P80005	Adora	1990	NETH	Primura × Alcmaria	Fresh consumption
P80006	Adretta	1975	GER	LU. 59.884/3 × Axilia	Fresh consumption
P80007	Agata	1990	NETH	BM 52-72 × Sirco	Fresh consumption
P80008	Agria	1985	GER	Quarta × Semlo	Processing industry
P80009	Ajiba	1992	NETH	AMINCA × VE 70-9	Fresh consumption
P80010	Albion	1895	NETH	REICHSKANZLER × SIMSON	Ancient cultivar
P80012	Allure	1990	NETH	Astarte × AM 66-42	Starch industry
P80014	Almera	1999	NETH	BM 77-2102 × AR 80-31-20	Fresh consumption
P80015	Alpha	1925	NETH	Paul Kruger × Preferent	Ancient cultivar
P80016	Am 66-42	/	NETH	VTN 62-33-3 × MPI 19268	Progenitor clone
P80018	Am 78-3704	/	NETH	AM 72-3477 × AM 70-2166	Progenitor clone
P80019	Amorosa	2000	NETH	Arinda × Impala	Fresh consumption
P80020	Ampera	1998	GER	AGUTI × PONTO	Fresh consumption
P80021	Amyla	1999	FRA	PROMESSE × ELEMENT	Starch industry
P80022	Anosta	1975	NETH	Ostara × Provita	Processing industry
P80024	Arcade	1999	NETH	AGRIA × VK 69-491	Fresh consumption
P80025	Arinda	1993	NETH	Vulkano × AR 74-78-1	Fresh consumption
P80026	Arnova	1999	NETH	Obelix × AR 76-168-1	Fresh consumption
P80028	Arran Chief	1911	UK	Paterson’s Victoria × Sutton’s Flourball	Ancient cultivar
P80030	Arran Victory	1918	UK	ABUNDANCE × ABUNDANCE	Ancient cultivar
P80031	Arrow	2004	NETH	Solara × Fresco	Fresh consumption
P80032	Astarte	1976	NETH	RR 62-5-43 × VTN 62-69-5	Starch industry
P80033	Asterix	1991	NETH	Cardinal × VE 70-9	Processing industry
P80034	Atlantic	1976	USA	Wauseon × Lenape	Processing industry
P80035	Aurora	1972	NETH	Profijt × AM 54-10	Starch industry
P80036	Ausonia	1981	NETH	Wilja × KONST 63-665	Fresh consumption
P80037	Avenance	/	NETH	Mercury × Florijn	Starch industry
P80038	Ballydoon	1931	UK	Herald × British Queen	Ancient cultivar
P80039	Bartina	1988	NETH	Saturna × ZPC 62-75	Fresh consumption
P80041	Bellini	2001	NETH	Mondial × Felsina	Fresh consumption
P80042	Berber	1984	NETH	Alcmaria × Ropta P 365	Fresh consumption
P80043	Bildtstar	1984	NETH	Winda × Saturna	Fresh consumption
P80044	Bintje	1910	NETH	Munstersen × Jaune d’or (=Fransen)	Fresh consumption
P80045	Biogold	2004	NETH	Novita × HZ 87 P 200	Processing industry
P80046	British Queen	1894	UK	PATERSON`S VICTORIA × BLUE DON	Ancient cultivar
P80047	Caesar	1990	NETH	Monalisa × Ropta B 1178	Processing industry
P80049	Charlotte	1981	FRA	Hansa × Danae	Fresh consumption
P80050	Cherie	1997	FRA	Roseval × AR 76-199-3	Fresh consumption
P80051	Cilena	1981	GER	Gelda × Hirla	Fresh consumption
P80052	Civa	1960	NETH	(BINTJE × (SASKIA × FRÜHMOLLE)) × CIV 49-901	Fresh consumption
P80053	Clivia	1962	GER	Seedling 1 × Seedling 17	Progenitor clone
P80055	Craigs Bounty	1946	UK	unknown	Ancient cultivar
P80056	Craigs Defiance	1938	UK	Epicure × Pepo	Ancient cultivar
P80057	Daisy	1998	FRA	GIPSY × CULPA	Fresh consumption
P80058	Deodara	1913	GER	Deutsches Reich × Jubel	Ancient cultivar
P80059	Désirée	1962	NETH	Urgenta × Depesche	Fresh consumption
P80060	Di Vernon	1922	UK	unknown	Ancient cultivar
P80061	Diamant	1982	NETH	Cardinal mutant	Fresh consumption
P80062	Ditta	1989	AUT	BINTJE × QUARTA	Fresh consumption
P80063	Donald	1996	NETH	Amera × W 72-19-443	Fresh consumption
P80064	Doon Star	1926	UK	Templar seedling × Majestic	Ancient cultivar
P80065	Dorado	1995	NETH	Spunta × VK 69-491	Processing industry
P80066	Doré	1947	NETH	DUKE OF YORK × BIERMA A 7	Ancient cultivar
P80067	Dr Mcintosh	1944	UK	(phu × HERALD) × HERALD	Ancient cultivar
P80068	Draga (1970)	1970	NETH	SVP 50-2017 × MPI 19268	Fresh consumption
P80069	Eersteling	1891	UK	Early Primrose × King Kidney	Ancient cultivar
P80070	Early Rose	1867	USA	Garnet Chili seedling	Ancestor
P80072	Eden (2000)	2000	FRA	EOLE × PENTLAND DELL	Fresh consumption
P80073	Ehud	1965	NETH	Panther × Karna 149	Starch industry
P80074	Eigenheimer	1893	NETH	Blaue Riesen × Fransen (Jaune d’or)	Ancestor
P80075	Elisabeth	2002	NETH	VE 82-96 × CILENA	Fresh consumption
P80076	Eos	2000	NETH	MONDIAL × W 72-22-496	Fresh consumption
P80077	Epicure	1897	UK	Magnum Bonum × Early Regent	Ancient cultivar
P80078	Escort	1982	NETH	RENTAL × CEB 64-197-16	Fresh consumption
P80079	Estima	1973	NETH	NOPOL × G 3014	Fresh consumption
P80080	Exquisa	1992	GER	Sigma × Ilse	Fresh consumption
P80081	Fabula	1997	NETH	Monalisa × Hudson	Fresh consumption
P80082	Felsina	1992	NETH	Morene × Gloria	Processing industry
P80083	Festien	2000	NETH	KARTEL × KA 80-1920	Starch industry
P80084	Fianna	1987	NETH	KONST 62-660 × AM 64-2	Processing industry
P80085	Fichtelgold	1945	GER	[(Zwickauer Fühe × Jubel) × Klara] × Mittelfrühe	Ancient cultivar
P80087	Flourball (Sutton’s)	1870	UK	unknown	Ancestor
P80088	Fontane	1999	NETH	Agria × AR 76-34-3	Processing industry
P80089	Fresco	1985	NETH	CEB 60-15-28 × PROVITA	Processing industry
P80090	Frieslander	1990	NETH	Gloria × 74 A 3	Fresh consumption
P80092	Furore	1930	NETH	Rode Star × Alpha	Ancient cultivar
P80093	Gladstone	1932	UK	ARRAN CHIEF × (MAJESTIC × GREAT SCOT)	Ancient cultivar
P80094	Gloria	1972	GER	Amex × Feldeslohn	Fresh consumption
P80095	Golden Wonder	1906	UK	Seedling of Early Rose	Ancient cultivar
P80096	Goya (2000)	2000	NETH	AM 78-4102 × KARDAL	Starch industry
P80097	Great Scot	1909	UK	Imperator × Champion	Ancient cultivar
P80098	Hansa	1957	GER	OBERARNBACHER FRÜHE × FLAVA	Fresh consumption
P80099	Herald	1928	UK	MAJESTIC × ABUNDANCE	Ancient cultivar
P80100	Hermes	1973	AUT	DDR 5158 × SW 163/55	Processing industry
P80102	Home Guard	1943	UK	DOON PEARL × CUMNOCK	Ancient cultivar
P80104	Impala	1989	NETH	52/72/2206 (BM 52-72) × BIRANCO	Fresh consumption
P80106	Innovator	1999	NETH	Shepody × RZ 84-2580	Processing industry
P80107	Inova	1999	NETH	NICOLA × IMPALA	Fresh consumption
P80110	Jaerla	1969	NETH	Sirtema × MPI 19268	Fresh consumption
P80113	Karnico	1987	NETH	Astarte × AM 66-42	Starch industry
P80114	Kartel	1994	NETH	KA 77-0133 × AM 78-3736	Starch industry
P80115	Katahdin	1932	USA	USDA 40568 × USDA 24642	Ancient cultivar
P80116	Kennebec	1948	USA	USDA B 127 × USDA 96-56	Ancient cultivar
P80117	Kepplestone Kidney	1900	UK	unknown	Ancient cultivar
P80118	Kerpondy	1949	FRA	unknown	Ancient cultivar
P80119	Kerr’s Pink	1907	UK	Fortyfold × (Abundance or Smiths early)	Ancient cultivar
P80121	Kondor	1984	NETH	KONST 61-333 × WILJA	Fresh consumption
P80122	Kuras	1996	NETH	BRDA (=PG 285) × VK 69-491	Starch industry
P80123	Kuroda	1998	NETH	AR 76-199-3 × KONST 80-1407	Fresh consumption
P80124	Lady Christl	1996	NETH	WS 73-3-391 × Mansour	Fresh consumption
P80125	Lady Claire	1996	NETH	Agria × KW 78-34-470	Processing industry
P80126	Lady Olympia	1996	NETH	AGRIA × KW 78-34-470	Processing industry
P80127	Lady Rosetta	1988	NETH	CARDINAL × VTN 62-33-3	Processing industry
P80128	Laura	1998	GER	Rosella × L 6140/2	Fresh consumption
P80129	Lenape	1967	USA	USDA B 3672-3 × Delta Gold	Fresh consumption
P80130	Leyla	1988	GER	7338/812 × CULPA	Fresh consumption
P80132	Liseta	1988	NETH	Spunta × VE 66-295	Fresh consumption
P80134	Majestic	1911	UK	unknown breeding line × British Queen	Ancestor
P80135	Marfona	1977	NETH	PRIMURA × KONST 51-123	Fresh consumption
P80137	Maritiema	1991	NETH	AM 71-125 × VE 70-9	Processing industry
P80139	Markies	1997	NETH	FIANNA × AGRIA	Fresh consumption
P80140	May Queen	1890	UK	unknown	Ancient cultivar
P80141	Mercator	1999	NETH	KARTEL × KA 86-0008	Starch industry
P80142	Monalisa	1982	NETH	Bierma A 1-287 × Colmo	Fresh consumption
P80143	Mondial	1987	NETH	SPUNTA × VE 66-295	Fresh consumption
P80144	Morene	1983	NETH	RENOVA × AM 66-42	Processing industry
P80145	Mpi 19268	/	GER	S. demissum × Deodara	Progenitor clone
P80146	Myatt’s Ashleaf	1804	UK	unknown	Ancient cultivar
P80147	Nicola	1973	GER	CLIVIA × 6430/101	Fresh consumption
P80149	Niska	1990	USA	WISCHIP × LENAPE	Processing industry
P80150	Noisette	1993	FRA	AMINCA × PIROSCHKA	Fresh consumption
P80151	Nomade	1995	NETH	Elles × AM 78-3704	Starch industry
P80152	Noordeling	1928	NETH	BRAVO × JAM	Ancient cultivar
P80153	Obelix	1988	NETH	Ostara × Renska	Fresh consumption
P80155	Pallas	2003	NETH	KW 84-11-220 × VDW 85-72	Processing industry
P80158	Peerless	1862	USA	Garnet Chili seedling	Ancient cultivar
P80159	Pentland Dell	1961	UK	Roslin Chania × Roslin Sasamua	Processing industry
P80160	Pepo (1919)	1919	GER	Deutsches Reich × Jubel	Ancient cultivar
P80161	Picasso	1994	NETH	Cara × Ausonia	Fresh consumption
P80162	Première	1979	NETH	Civa × Provita	Processing industry
P80163	Prevalent	1966	NETH	AMBASSADEUR × LOMAN M 54-106-1	Fresh consumption
P80164	Primura	1961	NETH	Sirtema × Majestic	Fresh consumption
P80165	Profijt	1949	NETH	Prummel K 264 × Matador	Ancient cultivar
P80166	Ramos	2000	NETH	AGRIA × VK 69-491	Fresh consumption
P80167	Record	1932	NETH	Trenctria × Energie	Ancient cultivar
P80169	Red Scarlett	1999	NETH	ZPC 80-239 × IMPALA	Fresh consumption
P80170	Redstar	1997	NETH	Bildstar × VDW 76-30	Processing industry
P80172	Remarka	1991	NETH	Edzina × AM 66-42	Processing industry
P80173	Romano	1981	NETH	DRAGA × DÉSIRÉE	Fresh consumption
P80175	Russet Burbank	1908	USA	Mutant of Burbank	Processing industry
P80176	Samba	1989	FRA	ROSEVAL × BARAKA	Fresh consumption
P80177	Santana	1994	NETH	Spunta × VK 69-491	Processing industry
P80178	Santé	1983	NETH	Y 66-13-636 × AM 66-42	Fresh consumption
P80179	Saskia	1946	NETH	Rode Eersteling × Herald	Fresh consumption
P80180	Saturna	1964	NETH	Maritta × (Record × CPC 1673-1adg)	Processing industry
P80181	Seresta	1994	NETH	AM 78-3704 × Sonata	Starch industry
P80182	Shamrock	1900	IRL	unknown	Ancient cultivar
P80183	Shepody	1980	CAN	Bake King × F58050	Processing industry
P80184	Sirtema	1947	NETH	DORST H 123A × FRUHMOLLE	Fresh consumption
P80185	Spunta	1968	NETH	Bea × USDA 96-56	Fresh consumption
P80186	Sunrise	1984	USA	Wauseon × USDA B 6563-2	Processing industry
P80187	Tahi	1960	NZL	SEBAGO × HARFORD	Fresh consumption
P80188	Tasso	1963	GER	seedling × Biene	Processing industry
P80189	The Alness	1934	UK	ABUNDANCE × MAJESTIC	Ancient cultivar
P80190	Timate	1984	NETH	Elvira × AM 66-42	Fresh consumption
P80191	Tinwald’s Perfection	1914	UK	unknown	Ancient cultivar
P80192	Toyoshiro	1976	JPN	HOKKAI 19 × ENIWA	Fresh consumption
P80193	Triplo	2000	NETH	AGRIA × FRESCO	Processing industry
P80194	Triumf	1921	NETH	Eigenheimer × Cimbals Neue Imperator	Ancient cultivar
P80196	Ulster Glade	1961	UK	ULSTER EMBLEM × adg-hybrid	Fresh consumption
P80197	Ulster Knight	1954	UK	CLARKE 736 × CRAIGS DEFIANCE	Fresh consumption
P80198	Ulster Sceptre	1962	UK	Pentland Ace × Ulster Prince	Fresh consumption
P80199	Ultimus	1935	NETH	Rode star × Pepo	Ancient cultivar
P80200	Umatilla Russet	1998	USA	Butte × A 77268-4	Fresh consumption
P80201	Up To Date	1894	UK	Patersons Victoria × Blue Don	Ancient cultivar
P80202	Urgenta	1951	NETH	FURORE × KATAHDIN	Fresh consumption
P80203	Usda 96-56	/	USA	USDA 3895-13 × EARLAINE	Progenitor clone
P80204	Ve 66-295	/	NETH	Amelio × HVT 60-8-3	Progenitor clone
P80205	Ve 70-9	/	NETH	Alcmaria × VTN 62-33-3	Progenitor clone
P80206	Ve 71-105	/	NETH	AM 67-136 × AM 67-59	Progenitor clone
P80207	Ve 74-45	/	NETH	Sinaeda × AM 66-42	Progenitor clone
P80208	Victoria	1997	NETH	AGRIA × ROPTA J 861	Processing industry
P80209	Virgo	2002	NETH	NICOLA × AM 78-3704	Fresh consumption
P80210	Vivaldi	1998	NETH	TS 77-148 × MONALISA	Fresh consumption
P80211	Vk 69-491	/	NETH	VK 64-56 × VTN 62-33-3	Progenitor clone
P80212	Voran	1931	GER	Kaiserkrone × Spatgold of Herbstgelbe	Ancient cultivar
P80213	Voyager	2003	NETH	RZ 85-238 × OBELIX	Processing industry
P80214	Vtn 62-33-3	/	NETH	((V 24/20 × ULSTER KNIGHT)1 × PROFIJT)15 ×	Progenitor clone
				(VRN I-3 × PROFIJT)5	
P80215	W 72-22-496	/	NETH	REDBAD × Y 66-13-636	Progenitor clone
P80216	Wauseon	1967	USA	USDA B 4159-8 × KATAHDIN	Fresh consumption
P80217	Wilja	1967	NETH	CLIMAX × KONST 51-123	Fresh consumption
P80218	Winston	1992	UK	KISMET × DXMP 70	Fresh consumption
P80219	Wisent	2005	NETH	Prudenta × Karakter	Starch industry
P80220	Y 66-13-636	/	NETH	Y 62-2-221 × AMARYL	Progenitor clone
P80221	Yam	1787	UK	unknown	Ancient cultivar
P80222	Yukon Gold	1980	CAN	NORGLEAM × USW 5279-4	Processing industry

## Appendix D. Acronyms for Traits Included in the Analysis

**Trait**	**Description**	**Units**
*tm1*	Inflection point in the Phase 1: Buildup of canopy development	td
*t1*	Period from plant emergence to maximum soil coverage	td
*t2*	Initiation of senescence or Phase 3	td
*te*	Total growing period till canopy is dead	td
*Vx*	Maximum %SC reached	%
*Cm*	Maximum progression rate of soil coverage during Phase 1	%SC/td
*AP1*	Area under canopy cover curve for Phase 1	%SC.td
*AP2*	Area under canopy cover curve for Phase 2	%SC.td
*AP3*	Area under canopy cover curve for Phase 3	%SC.td
*AUC*	Total area under the canopy curve	%SC.td
*t2–t1*	Duration of Phase 2	td
*te–t2*	Duration of Phase 3	td
Y_DM	Tuber dry matter	g/m^2^
DM%	Tuber dry matter percentage	%
[N]	Tuber nitrogen concentration	g/kg
NUpt	Tuber nitrogen uptake	g/m^2^
TbNUE	Tuber nitrogen use efficiency	g/g
TbndMX	Maximum tuber number	Tb #/m^2^
TbndB	Tuber size having the maximum tuber number	mm
TbndA	Tuber number dispersion parameter	-
TbwdMX	Maximum tuber weight	g/m^2^
TbwdB	Tuber size having the maximum tuber weight	mm
TbwdA	Tuber weight dispersion parameter	-
mt_as	Maturity assessment (values 3 to 8)	-
